# Growth Patterns in Seedling Roots of the Pincushion Cactus *Mammillaria* Reveal Trends of Intra- and Inter-Specific Variation

**DOI:** 10.3389/fpls.2021.750623

**Published:** 2021-10-08

**Authors:** José de Jesús González-Sánchez, Itzel Santiago-Sandoval, José Antonio Lara-González, Joel Colchado-López, Cristian R. Cervantes, Patricia Vélez, Jerónimo Reyes-Santiago, Salvador Arias, Ulises Rosas

**Affiliations:** ^1^Jardín Botánico, Instituto de Biología, Universidad Nacional Autónoma de México, Mexico City, Mexico; ^2^Posgrado en Ciencias Biológicas, Universidad Nacional Autónoma de México, Mexico City, Mexico; ^3^Departamento de Botánica, Instituto de Biología, Universidad Nacional Autónoma de México, Mexico City, Mexico

**Keywords:** Cactaceae, natural variation, root architecture, succulent plant, plant evolution, root development, evo-devo, microevolution

## Abstract

Genetic mechanisms controlling root development are well-understood in plant model species, and emerging frontier research is currently dissecting how some of these mechanisms control root development in cacti. Here we show the patterns of root architecture development in a gradient of divergent lineages, from populations to species in *Mammillaria*. First, we show the patterns of variation in natural variants of the species *Mammillaria haageana*. Then we compare this variation to closely related species within the Series *Supertexta* in *Mammillaria* (diverging for the last 2.1 million years) in which *M. haageana* is inserted. Finally, we compared these patterns of variation to what is found in a set of *Mammillaria* species belonging to different Series (diverging for the last 8 million years). When plants were grown in controlled environments, we found that the variation in root architecture observed at the intra-specific level, partially recapitulates the variation observed at the inter-specific level. These phenotypic outcomes at different evolutionary time-scales can be interpreted as macroevolution being the cumulative outcome of microevolutionary phenotypic divergence, such as the one observed in *Mammillaria* accessions and species.

## Introduction

A long standing debate in evolutionary biology is whether the nature of macroevolutionary change can be explained based on the principles and processes of microevolution. One possibility is that the macroevolutionary outcomes are the result of the cumulative microevolutionary processes, so the footprint of microevolution can be seen at higher levels of taxonomic divergence. This possibility has been tested in some organisms such as crocodiles, in which intraspecific crane variation (a highly robust trait) spans half of the extant species (Okamoto et al., 2015). Furthermore, in model species such as *Drosophila*, it has been experimentally shown that the genetic variation explaining divergent pigmentation patterns among species, are shared with the genetic variation displayed within species (Wittkopp et al., 2009). On the other hand, it has been argued that morphological divergence between species is often non-adaptive, as compared to variation within species. This is because regardless of their adaptive value, phenotypic differentiation has been suggested to be frequently rapid, and random in direction, involving the evolution of gene regulation, pleiotropy, epistasis and canalization (Davis and Gilmartin, 1985), which in turn could result in different nature of the variation within and between species. Despite the relevance of the question for the understanding of evolution and development of breeding strategies, in plants, to our knowledge there are very few comprehensive cases where these ideas have been tested at the morphological or genetic level. One of the few examples is the case of cacti, in which the comparison of micro- vs. macro-evolutionary divergence has been indirectly addressed in the determinate primary growth of the root apex, a highly conserved trait in the subfamily Cactoideae (Shishkova et al., 2013; Rodriguez-Alonso et al., 2018) in which the timeframe of this apex determination is correlated with environmental factors, within and between species (Martino et al., 2018); however, the number of species and accessions are low to draw conclusions about the nature of evolutionary divergence. Therefore, we attempt to provide elements to this discussion in plant evolution, studying the root development of *Mammillaria* species.

*Mammillaria* is the most diverse genus within the Cactaceae family. It comprises 155–320 species mainly distributed in Mexico (Reppenhagen, 1992; Guzmán et al., 2003; Hunt et al., 2006; Hernández and Gómez-Hinostrosa, 2015; Villaseñor, 2016). The genus is characterized by plants with tubercles arranged in spiraled rows, the areola is dimorphic, that is, one part is at the base from where the flowers, bristles or branches arise, and another at the tip of the tubercles where spines grow (Bravo and Sánchez-Mejorada, 1991; Scheinvar, 2004). It has been proposed that *Mammillaria* s.l. is non-monophyletic (Butterworth and Wallace, 2004), and recently it was also proposed that the Mammiloid clade circumscribes three monophyletic genera: *Mammillaria* s.s., *Coryphantha* and *Cochemiea* s.l. (Breslin et al., 2021). The Mammilloid clade is estimated to have diverged for the last 8.62 million years (Hernández-Hernández et al., 2014). In addition, the *M. haageana* genome size has been estimated to be 1 C = 1.5 Gbp (Christian et al., 2006), and a plastid genome of 115 kbp (Hinojosa-Alvarez et al., 2020). Despite our current incomplete understanding of the phylogenetic relationships among *Mammillaria* species, a core *Mammillaria* set of species grouped into 8 subgenres and 16 series have been proposed (Butterworth and Wallace, 2004; Hernández and Gómez-Hinostrosa, 2015). Most species are distributed in arid or semi-arid lands, but some species are also found in deciduous forests, or even in oak-pine forests (Hernández and Gómez-Hinostrosa, 2015).

Among the series, *M*. ser. *Supertextae* is characterized by the presence of cuticular crystals (Lüthy, 1995) and flowers smaller than 15 mm (Hunt et al., 2006). The species that make up the series are distributed from Central Mexico to Central America (Pilbeam, 1999). It has been suggested that the sister series of *M*. ser. *Supertextae* is *M*. ser. *Polyachanthae*, supported by a deletion in *rpl16*; it was also found that *M*. ser. *Supertextae* started diverging about 2.1 million years ago. According to an accepted classification (Hunt, 1983; Hunt et al., 2006), the *Supertextae* series comprises 9 species: *M. albilanata* Backeb., *M. crucigera* Mart., *M. columbiana* Salm-Dyck, *M. dixanthocentron* Backeb. ex Mottram., *M. flavicentra* Backeb., *M. haageana, M. halbingeri* Boed., *M. huitzilopochtli* D.R.Hunt, and *M. supertexta* Mart. Ex Pfeiff. Within the *Supertextae* Series, *M. haageana* is a highly variable species, which seems to have a complex evolutionary history resulting in an ample distribution along the Mexican neovolcanic axis, inhabiting a wide range of environments from pine-oak forests to shrubs and deserts. These locations have been classified into subspecies according to their distribution, plant shape, spination patterns, flower color, among other traits (Guzmán et al., 2003). *M. haageana* is a highly charismatic species as ornamental, and it is one of the few cacti species to have been reported by the early expeditions to the New World of Sessé & Mociño during the XVIII Century (Mociño and Sessé, 2015). Currently it is one of the most representative flagships of the UNAM Jardín Botánico for conservation efforts. Despite its biodiversity, horticultural, historic and conservation importance, the evolutionary history of *M. haageana* is far from being fully understood. Thus, in this work we refer to the *M. haageana* natural variants as accessions.

In sessile organisms such as plants, resource foraging by roots, allocation of assimilates and growth are complex problems vital to maximize survival and reproductive success. Evolutionary processes have generated and tested biological trade-offs by optimizing urgent tasks, while allocating fewer resources to other non-imperative tasks. One could consider that species and populations are optimal to multitask in their native environment; however, their optimality is constrained by the previous best solutions for different tasks. During plant development some of the most imperative tasks that roots perform, and particularly for desert plants, are water uptake and nutrient foraging. This is why plants must decide how to grow to optimize resource uptake, but also some of these growth strategies might be fixed to maximize fitness. We currently have a comprehensive understanding of the molecular mechanisms controlling growth and drought stress responses in model plants. The challenge is to understand the genetic mechanisms on how desert plants uptake resources particularly by roots, grow and develop, in early stages when seedlings are highly sensitive to mortality.

In this work we present a comprehensive picture on how roots from the *Mammillaria* genus grow during early stages of development (first few months). We used three groups of *Mammillaria* stocks (*Mammillaria* species, *M*. ser. *Supertextae* species, and *M. haageana* accessions) representing an ample range of evolutionary divergence (up to 8 million years), and used this framework to ask the question whether natural variation recapitulates the diversity between species, and test the hypothesis whether microevolutionary phenotypic evolution resemble that from macroevolutionary processes.

## Materials and Methods

### Plant Material

**Figure 1:**
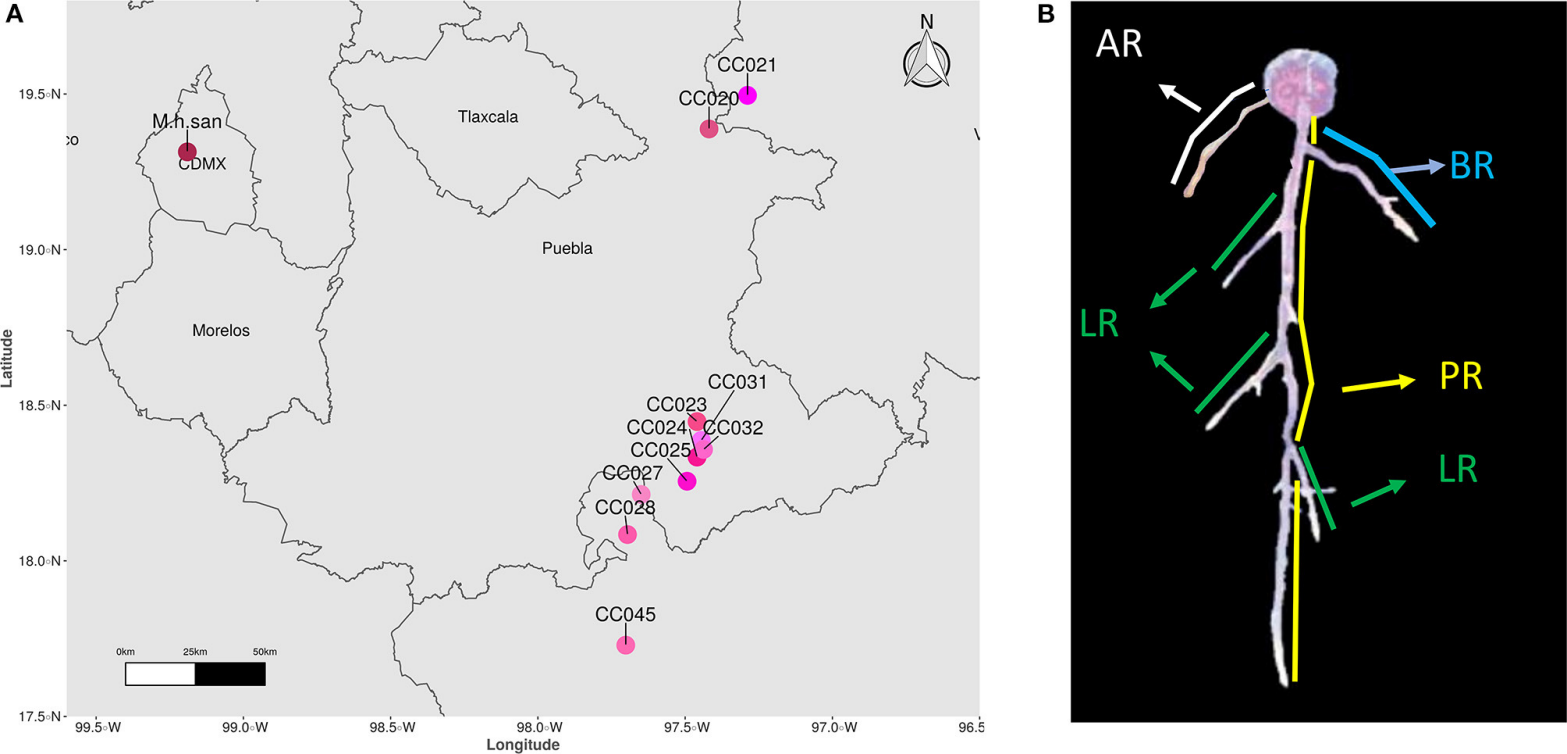
**(A)** Geographical origin of *M. haageana* accessions. **(B)** Characterization of root attributes in a *Mammillaria* plant. PR, Principal Root; LR, Lateral Root; BR, Basal Root; AR, Adventitious Root.

Seeds from *Mammillaria* species were obtained from the Jardín Botánico (UNAM) collections, harvested from the living cacti collections within 1–3 years prior germination. To represent species from the *Mammillaria* genus, we selected 16 species belonging to 8 series. For simplicity, acronyms of these species were created as follows: *M. carnea* Zucc. ex Pfeiff. (*M. car*), *M. coahuilensis* (Boed.) Moran (*M. coa*), *M. duwei* Rogoz. and P. J. Braun (*M. duw*), *M. formosa* Scheidw. (*M. for*), *M. hernandezii* Glass and R. A. Foster (*M. her*), *M. karwinskiana* Mart. (*M. kar*), *M. lasiacantha* Engelm. (*M. las*), *M. magnimamma* Haw. (*M. mag*), *M. pectinifera* F. A. C. Weber (*M. pec*). For *M*. ser. *Supertextae* species we could only obtain 7 species, and they were abbreviated as follow: *M. albilanata* (*M. alb*), *M. crucigera* (*M. cru*), *M. dixanthocentron* (*M. dix*), *M. flavicentra* (*M. fla*), *M. huitzilopochtli* (*M. hui*), and *M. supertexta* (*M. sup*). As for the *M. haageana* accessions, seeds were collected from the wild in 2018 (Figure 1A), assigned accession numbers according to our previous work (Cervantes et al., 2021), and their corresponding plants were deposited in the Jardín Botánico (Instituto de Biología, UNAM) collection (collection license SGPA/DGGFS/712/3690/10). For *M. haageana* subspecies *san-angelensis* (*M. h. san*) seeds were obtained from the Adoption Center Conservation Program for Endangered Species at Jardín Botánico (Instituto de Biología, UNAM).

### Growth Conditions and Phenotyping

Seeds were disinfected by a wash in 70% commercial bleach for 5 mins, followed by three washes in sterile distilled water, within a laminar flow-hood with HEPA filter (Veco, México). The seeds were suspended in 0.1% agar to facilitate their manipulation and adhesion to the sowing plate. Seeds were sown in 12 × 12 cm petri dishes (Greiner Bio-One, Cat 688102), with 75 mL of 50% strength Murashige-Skoog media (Caisson Labs, Cat MSP09-1LT), added with 0.05 MES salts (MP Biomedicals, Cat 152454), adjusted to a pH of 5.7, and solidified with 1% agar (Sigma Life Science, Cat A1296-1KG). Each plate was sown with 49 evenly spaced seeds (7 by 7 disposition), and germinated in a growth chamber (Percival Scientific, Cat CU22L) at 28°C with a 16/8 long day photoperiod, as in our previously published experimental set up (Rosas et al., 2021). Germination was recorded every third day, for 45 days, after which we had plenty of healthy seedlings with 40–45 days after germination, and that is why we chose this age for further procedures. The seedlings were then transplanted to fresh plates prepared as mentioned above, and arranged in two rows of 5 seedlings in each plate. To adhere the roots to the plate, drops of 0.3% agar were added to the root, and plates were horizontally kept for 3 days, after which plates were switched to vertical position, and plants were kept in the same growth chamber at 28°C and 16/8 photoperiod. Digital images from plates were obtained using a scanner (EPSON Perfection v600 Photo) at a 600 dpi resolution in JPG format, at 45, 73, 101, 129, and 157 days post germination, corresponding to periods of 4 weeks, so the differences were noticeable. From each species or *M. haageana* accession we obtained 20–40 plants, which were considered as biological replicates (Supplementary Table 1). Because of the magnitude of the experiment, these plants were obtained in sequential batches. We used the free software ImageJ (version 1.52a), coupled to a measuring system previously used to calculate the Rhizochron index (Colchado-López et al., 2019), and whose scripts calculated several attributes of the roots: Total Root Length (TRL), Principal Root Length (PRL), Total Lateral Root Length (TLRL), Adventitious Root Length (ARL), Basal Root Length (BRL), number of Lateral Roots (nLR), number of Adventitious Roots (nAR), and number of Basal Roots (nBR). We defined the principal root as the dominant root axis in the early stages (45 and 73 days), the lateral roots as any root branching out from another root, the adventitious roots as those originating from the shoot (the hypocotyl), and basal roots as those roots originating from the first millimeter from the root-shoot junction on the root side (Figure 1B). These measurements were done for individual plants over time, so it was always clear what type of root was being measured. All gathered data can be found in the Supplementary Data Sheet 1.

### Data Processing and Clustering Analysis

Modified range plots for all genotypes were created using the package “ggplot2,” in which the median value was expressed along with bars specifying the interquartile range, and atypic data points. For visualization purposes, all plots were done in log_10_ scale using the function “pseudo_log_trans()” from package “scales” to better appreciate the subtle differences toward low trait values in root variation. Cluster analysis for accession dissimilarity was performed on the medians of each group with euclidean distance, using the package “dendextend,” normalizing for trait variance. PCA analysis was performed by scaling the variables, using a Spearman correlation, and eigenvectors and eigenvalues were obtained with “eigen()” from “base.” Scree-plots and individual PCs boxplots were visualized using the “ggplot” and “ggrepel” packages, and the boxplots were aligned with the “plot_grid()” function from “cowplot.” Statistical differences for the PCs were analyzed with a Kruskal-Wallis test performed with the “kruskal()” function from “agricolae.” *Post-hoc* Dunn test was performed using the “dunnTest(),” from “FSA” package and letters were obtained using the “cldList()” function from “rcompanion.” All analyses were done in R version 4.0.1 (R Core Team, 2020).

## Results

### Natural Variation in Root Architecture Within *Mammillaria haageana* Accessions

**Figure 2:**
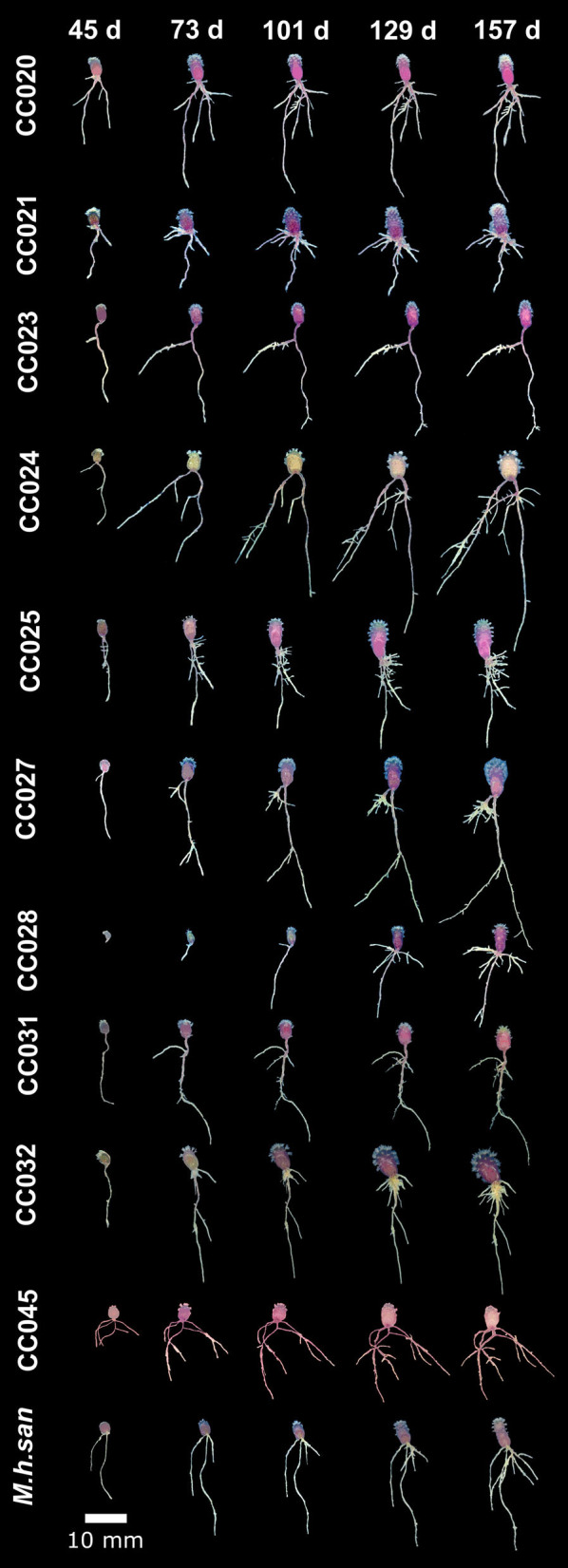
Growth dynamics in *M. haageana* accessions over five developmental stages (45, 73, 101, 129, and 157 days after germination).

To characterize the natural variation in developmental dynamics in a *Mammillaria* species, we chose *M. haageana*, a widely distributed species along the Mexican neovolcanic axis (Hunt et al., 2006; Arias et al., 2012), whose diversity we are currently characterizing (Cervantes et al., 2021), and we have established a collection of natural accessions at the Jardín Botánico (Instituto de Biología, UNAM). Using seeds from the wild, first we performed germination in aseptic conditions in order to study the variation in root architecture in natural accessions under a controlled environment. However, these germination efforts of *M. haageana* revealed the consistent recovery of a dematiaceous filamentous fungus emerging from seeds in some accessions originating from oak-pine forests, despite superficial decontamination of the material and its incubation under controlled conditions in growth chambers. We isolated this fungus for future characterizations and experiments (Supplementary Figure 1). However, our observations suggest that the presence of this fungus enhanced the suitable germination and posterior development of the early root system in contrast to seeds where this fungus was not present. This agrees with former work reporting seed germination in *Opuntia* depends on the presence of fungi to reduce mechanical resistance of the testa (Delgado-Sánchez et al., 2013). Furthermore, success in the propagation of cacti infected with fungi and bacteria has been documented, with *Mammillaria* spp. being more amenable (Fay and Gratton, 1992). However, our observations of plant-fungus interactions in *M. haageana* deserve further examination in future work. For the purpose of the current experiment, those contaminated *M. haageana* accessions had to be discarded from the analysis (CC022, CC030, and CC035), and from the 20 accessions that we currently have in the *M. haageana* collection, we were able to assess root growth dynamics in 11 of them (Figure 2).

**Figure 3:**
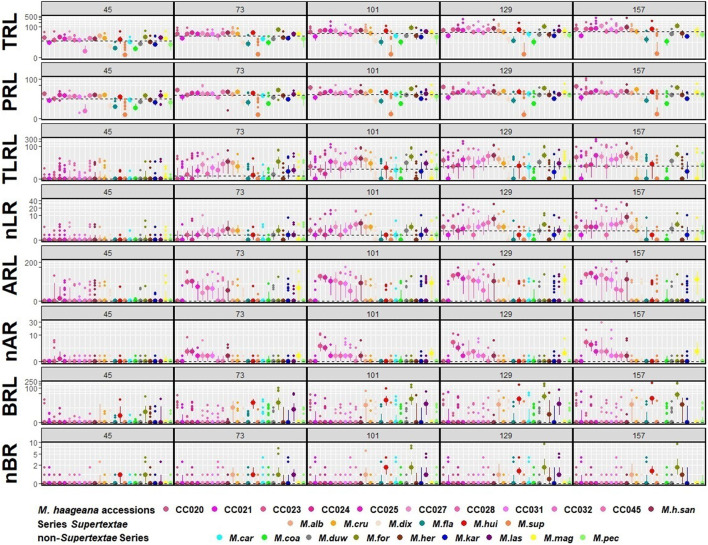
Quantitative dynamics of root growth in *M. haageana* accessions, *Supertextae* series *Mammillaria* species, and non-*Supertextae* series *Mammillaria* species, at 45, 73, 101, 129, and 157 days after germination. Large dots represent the median of the accession, smaller dots represent the outliers, and bars represent the interquartile range. Horizontal dotted-lines represent the overall median for the entire developmental stage. TRL, Total Root Length; PRL, Principal Root Length; TLRL, Total Lateral Root Length; ARL, Adventitious Root Length; BRL, Basal Root Length; nLR, number of Lateral Roots; nAR, number of Adventitious Roots; nBR, number of Basal Roots. *y* axes are expressed in base 10 pseudo-log scale.

Regarding the growth dynamics in *M. haageana*, despite being variants of the so-called same species, there is plenty of variation on the root architectural system when comparing different accessions (Figure 2), as well as within each accession (Figure 3), and different types of roots emerge at distinct time frames. In general, the lateral roots had a sustained growth during the first 101 days, but later it stagnated toward the 129 and 157 days after germination. Among accessions, the presence and elongation of lateral roots was highly variable over time, with emerging lateral roots in CC024 and CC032 at 45 days, but in other accessions they proliferated from day 73 onwards, except for the accession CC021 in which lateral roots were not visible before 101 days after germination.

Adventitious roots proliferated from day 73, and their growth was remarkable by day 129 and 157. However, in CC028 and CC031, a large proportion of the seedlings did not show adventitious roots before the day 129, suggesting that these two accessions have slower growth rates as compared to the rest of the accessions. Remarkably, CC024 and CC032 were the two accessions with the longest adventitious roots.

An interesting observation for *M. haageana* accessions is that in most seedlings we did not detect basal roots, however, in some exceptional plants, as in accessions CC021, CC025, and CC031, some adventitious roots emerged before the day 73, and elongated later on, but no new adventitious root emerged in later stages.

### Diversity in Root Architecture Between Closely Related *Mammillaria* Species

**Figure 4:**
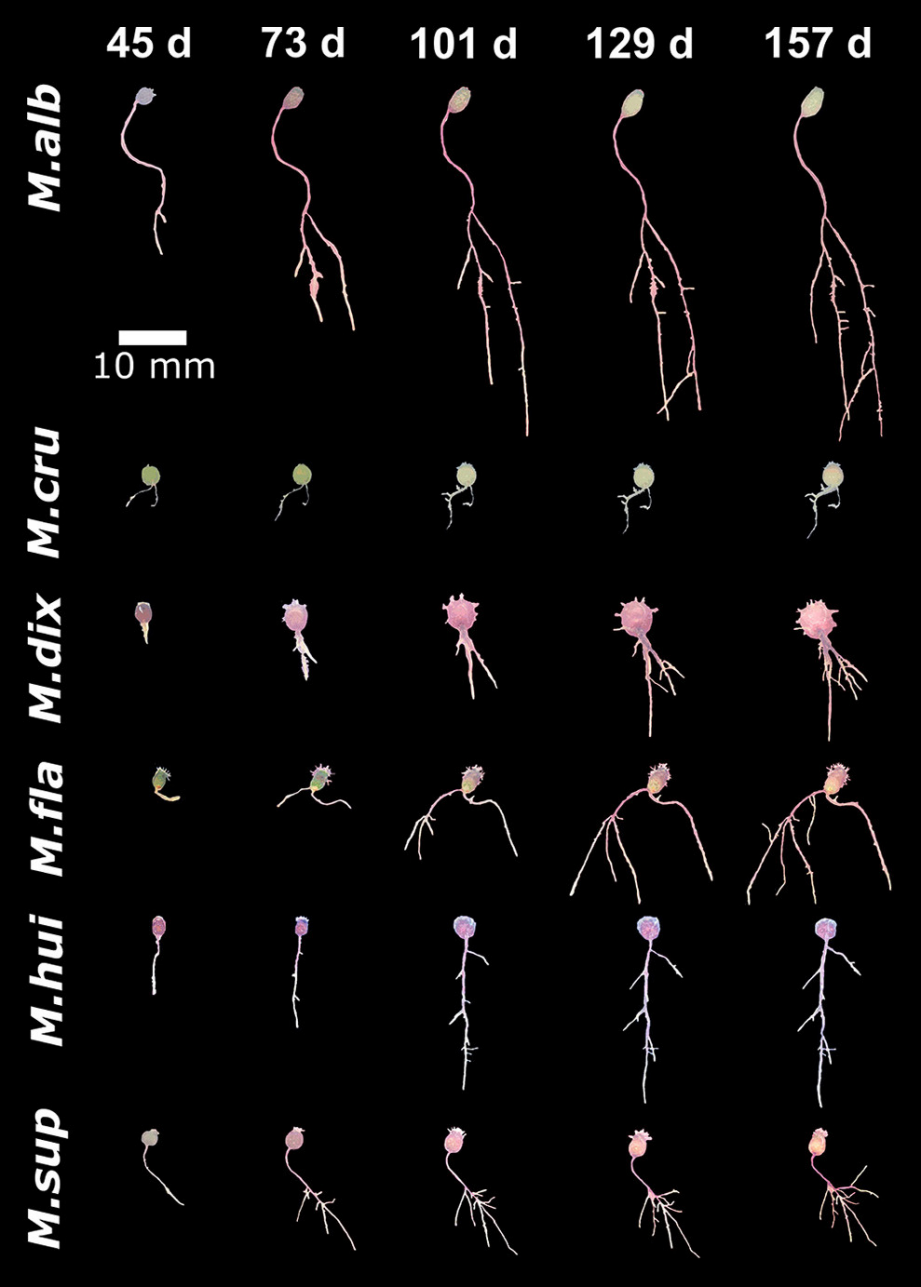
Growth dynamics in *Mammillaria* species from the Series *Supertextae* over five developmental stages (45, 73, 101, 129, and 157 days after germination). For simplicity, acronyms of these species were created as follow: *M.alb, M. albilanata*; *M.cru, M.crucigera*; *M.dix, dixanthocentron, M.fla, M. flavicentra*; *M. hui, M. huitzilopochtli*; *M.sup, M. supertexta*.

To compare the growth dynamics of *M. haageana* to the set of sister species within the series *Supertextae*, we grew seedlings of *M. flavicentra, M. dixanthocentron, M. albilanata, M. supertexta, M. huitzilopochtli*, and *M. crucigera*, in the same controlled conditions (Figure 4). When observing these phenotypes, we detected more dramatic differences between *Supertextae* series as compared to the *M. haageana* accessions (Figures 2–4). For instance, *M. cru* or *M. fla* had limited growth as opposed to the prominent root growth of *M. alb*. On the other hand, *M. alb* and *M. haageana* accessions had the longest root systems, as compared to the other *Supertextae* species. Different to the rest of the *Supertextae* species and *M. haageana* accessions, lateral roots in *M. alb* and *M. sup* did not slow down their growth toward the later stages of development.

A compelling observation is that most *Supertextae* species did not develop adventitious roots, as was the case of *M. alb, M. dix*, and *M. sup*. Some plants had adventitious roots in *M. cru, M. hui*, and *M. fla*. This pattern was somehow similar to what was observed in *M. haageana* accessions, which might suggest that the absence or poor growth of adventitious roots, might be a defining morphological feature of *Supertextae* species. In addition, very few individuals in most *Supertextae* species develop basal roots, suggesting that basal roots might also be a characteristic feature of the series.

### Diversity in Root Architecture Between *Mammillaria* Species

**Figure 5:**
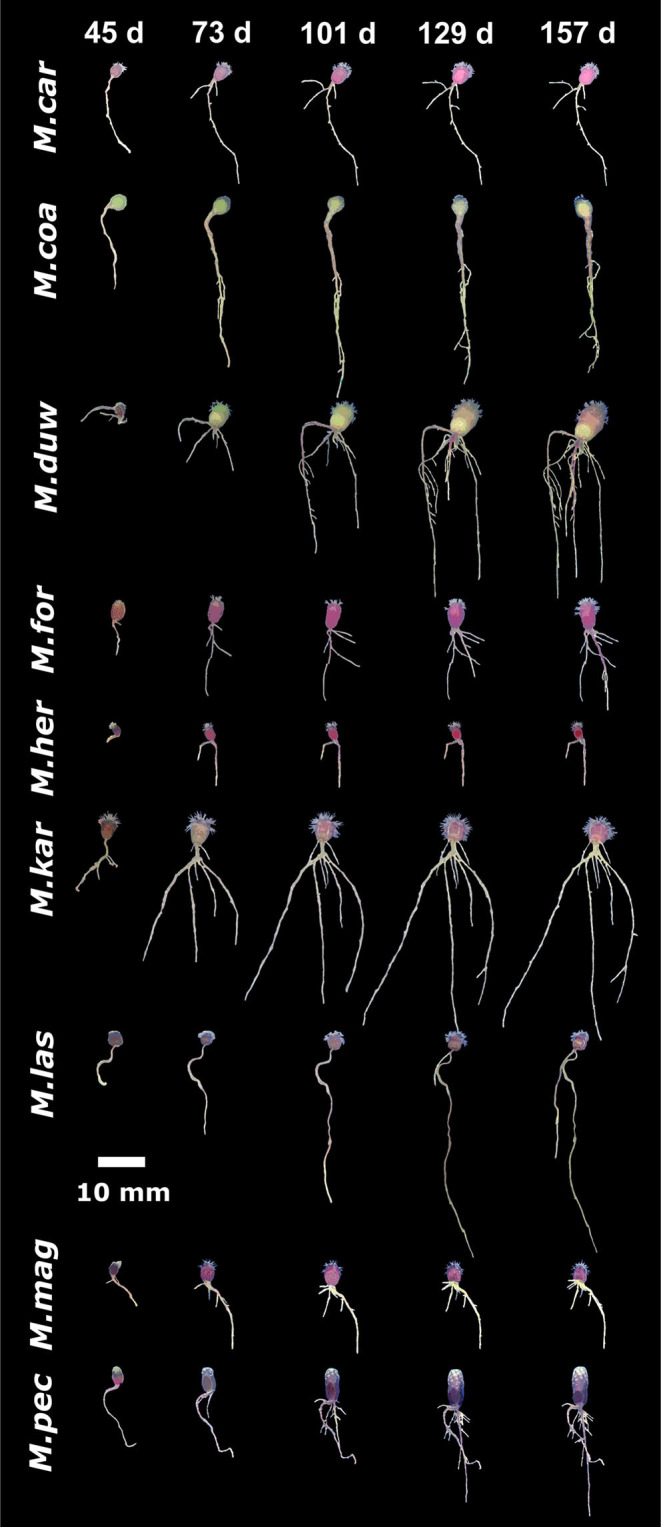
Growth dynamics in *Mammillaria* species from series other than *Supertextae*, over five developmental stages (45, 73, 101, 129, and 157 days after germination). For simplicity, acronyms of these species were created as follow: *M. car, M. carnea*; *M. coa, M. coahuilensis*; *M. duw, M. duwei*; *M. for, M formosa*; *M. her, M. hernandezi*; *M. kar, M. karwinskiana*; *M. las, M. lasiacantha*; *M. mag, M. magnimama*; *M. pec, M. pectinifera*.

To compare the natural variation in root architecture in *M. haageana* accessions and *Supertextae* species to a higher order of evolutionary divergence, we grew a set of non-*Supertexta* Series *Mammillaria* species (Figure 5). Similar to what occurred in *M. haageana* accessions and *Supertextae* species, the overall root architecture grows at a sustained rate, but slows down in the later stages of development. Additionally, when comparing individuals from the same species, some of them are highly variable (i.e., *M. kar*) while others are pretty robust in their overall root size (i.e., *M. her*). When comparing among species, the clearest differences in overall root size were observed at 129 days after germination, in which time *M. kar* has the highest values, while *M. her, M. car*, and *M. cru* have the lowest values.

Regarding the principal root, it has an accelerated growth during the first developmental stages but later stagnates toward the later stages. None of the species principal roots exceeded the 60 mm of length. However, the principal root was one of the attributes with large variation among individuals of the same species, such as in *M. duw* and *M. kar*, while variation is tighter in *M. her*. A similar pattern was observed for the attributes of lateral roots.

Basal roots were present in all species, which was different to what occurs in *M. haageana*, in which basal roots are rare. In non-*Supertextae* species, basal root proliferation and growth was constant, having *M. kar* and *M. duw* the highest values. However, when observing the presence and length of adventitious roots, most species lacked them, being *M. her, M. cru*, and *M. duw* the species with the outstanding values. Moreover, the emergence of adventitious roots seems to be a stochastic event, in which some of the individuals within a species develop these types of roots while others do not. This was the case of *M. her*, in which adventitious roots can be observed in 50% of the plants. These two attributes, the presence of basal and adventitious roots can be interpreted as strategies to cope with stresses, as we reported for the cacti species *Echinocactus platyacanthus* grown under salt conditions (Rosas et al., 2021); but the fact that not all plants present these types of roots could also be interpreted as a pre-established bet-hedging strategy to cope with challenging environmental circumstances, as has been shown for other type of traits in model organisms such as yeast (Levy et al., 2012).

### Comparing Trends of Natural Variation and Diversity in Root Architecture

**Figure 6:**
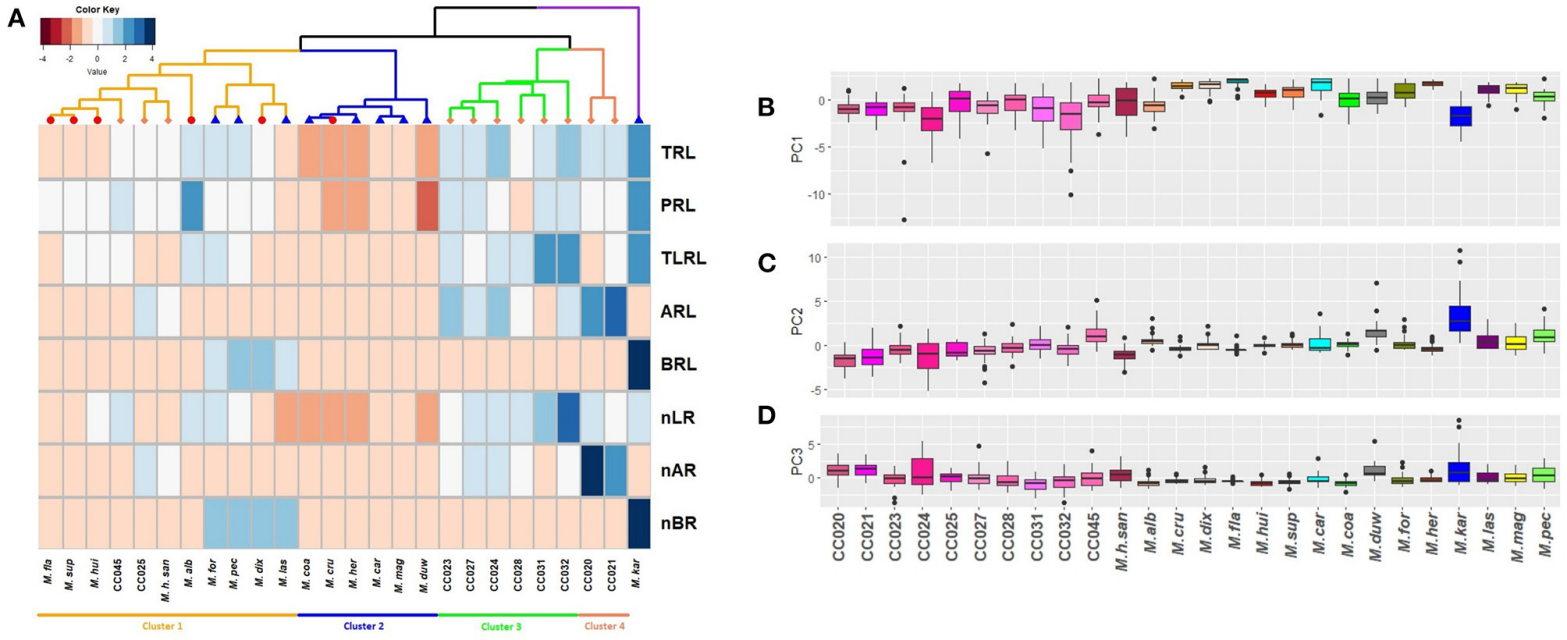
Trends of phenotypic variation at different evolutionary levels. **(A)** Clustering (Euclidean distance) of *Mammillaria* accessions according to their root architecture phenotypes at 129 days after germination developmental stage. On dendrogram, coral rhomboids represent *M. haageana* accessions, red circles represent *Supertextae* series species, and blue triangles represent non-*Supertextae* series species. TRL, Total Root Length; PRL, Principal Root Length; TLRL, Total Lateral Root Length; ARL, Adventitious Root Length; BRL, Basal Root Length; nLR, number of Lateral Roots; nAR, number of Adventitious Roots; nBR, number of Basal Roots. **(B–D)** Boxplots of each of the 3 Principal Components that together captured 90.6% of the variation. Significant differences are shown with letters.

In order to compare the trends of natural variation in *M. haageana* species, and the two levels of diversity in *Supertextae* Series and other non-*Supertextae* species, we performed a euclidean clustering analysis on the medians of eight root variables corresponding to TRL, PRL, TLRL, ARL, BRL, nLR, nAR, and nBR (Figure 6A). Performing the same clustering analysis using five root variables, excluding the number of different types of roots, gave a similar result (Supplementary Figure 2), recapitulating a similar clustering topography. We found four main clusters: the first cluster contains most *Supertextae* species (except for *M. cru*), as well as three non-*Supertextae* species (*M. for, M. pec*, and *M. las*); the cluster also includes three accessions of *M. haageana* (*M. h. san*, CC025 and CC045), which might be expected as *M. haageana* is one of the *Supertextae* species. Thus, this cluster shows a mixture of *Supertextae* species plus non-*Supertextae* species. The second cluster contains mostly non-*Supertextae* species in addition to *M. cru* which belongs to *Supertextae*, once again showing an asymmetric mixture *Supertextae* and non-*Supertextae* species. Interestingly, the third and fourth cluster grouped together most of the *M. haageana* accessions, leaving outside the cluster *M. h. san*, CC025, and CC045, which belonged to the first cluster. Within the fourth cluster, CC020 and CC021 grouped together, and this would be expected as these accessions were classified as *M. haageana* subsp. *haageana* (Guzmán et al., 2003), and their locations are within 10–12 km from one another. A similar case was observed on CC031 and CC032, grouping together in cluster three, which are classified as *M. haageana* subsp. *meissneri* (Guzmán et al., 2003), and whose populations are located 2 km away from one another. However, CC025 falls within cluster one, despite being within 5–6 kms from CC024 (in cluster three), both of them classified as *M. haageana* subsp. *meissneri*, but being CC025 more similar to *M. haageana* subsp. *san-angelensis*, which is located more than 230 km from those populations, inhabiting a completely different environment. Finally, *M. kar* does not cluster with any of the rest of the *Mammillaria* genotypes; this species is characterized by the prominent development of basal roots (Figure 4), which might be an adaptation to the environments where it is present. In addition, *M. kar* is the sister species of *M. car* (Butterworth and Wallace, 2004), and yet their root architecture was not as similar as it could be expected. However, *M. kar* has an ample distribution range, and it remains to be seen whether other *M. kar* accessions display phenotypic variation, similar to what we observed in *M. haageana*. An interesting observation is that, within the second cluster, which is dominated by non-*Supertextae* species, we found *M. cru*, which was not unexpected as *M. her* and *M. car* have a similar distribution as *M. cru*, mainly in the surroundings of the Tehuacan-Cuicatlan Valley (Arias et al., 2012; Hernández and Gómez-Hinostrosa, 2015), perhaps even in sympatry. The predominant rock type in this cited area is limestone, so it is possible that these species have similar adaptations to the substrate environmental conditions, reflected in their root architecture.

To double check this observation, we performed a Principal Component Analysis, using the above mentioned data (Figures 6B–D). We found that 3 PCs capture 90.6% of the variation, having PC1 40.6%, PC2 30.7%, and PC3 19.2% (Supplementary Figure 3). Each of the other 5 PCs individually captured <10% each, and therefore we did not further consider them. As for PC1, four root traits contributed to its variance (TRL, TLRL, nRL, and PRL), for PC2 the complementary root traits contributed to its variance (nRB, BRL, nRA, ARL), and the variance of PC3 was a mix of root trait contributions from our eight measured roots attributes (Supplementary Tables 2, 3). We then plotted each of the PC-calculated values according to our categories: *M. haageana* accessions, *Supertextae* species, and non-*Supertextae* species. We found that only PC1 was able to distinguish between these three levels of comparison and particularly *M. haageana* accessions from the other species. However, PC2, nor PC3 distinguish between the evolutionary categories, further confirming that the trends of variation between different levels of evolutionary divergence, partially overlap.

If microevolutionary processes recapitulate those of larger evolutionary time-scales, the accessible phenotypic space explored by natural variants within a species, might continuously overlap with the phenotypic space of closely related species, or distantly related species. This is, each of the species might find phenotypic solutions, but often finding the same solutions expressed in other closely related species; the phylogenetically closer the other species are, the more likely species are to find a similar solution. In our analysis, we found that cluster three and four distinguish most of the studied *M. haageana* accessions, showing that *M. haageana* might have found an exclusive phenotypic space for its root architecture; however, three of the accessions fell in cluster one, which in turn is dominated by *Supertextae* species, as well as having three non-*Supertextae* species. Meanwhile, one of the *Supertextae* species (*M. cru*), also fell within cluster two, which was dominated by non-*Supertextae* species. On the other hand, our PCA also showed a similar pattern, in which some trends of variation (i.e., PC1), allowed the distinction of the evolutionary categories, while in other PCs (i.e., PC2 and PC3) the distinction was not possible. This shows a reiterative partial overlap between the lower evolutionary hierarchy and its contiguous higher evolutionary hierarchy. In other words, during the evolutionary process and adaptation to novel environments, the root architecture phenotypes do not fall far from the evolutionary tree.

Despite our detailed root architecture characterization, it is possible that similar to *M. haageana*, other *Mammillaria* species with wide distribution such as *M.car, M.alb, M. kar, M. mag, M. las, M. for*, and *M. hui*, might have local variants, and therefore their root phenotypes are only one small sample of the range of phenotypes the species can have. Thus, a similar approach to what was performed in *M. haageana* accessions is necessary for a more robust interpretation.

## Discussion

What originates macroevolutionary diversity has been for a long time the subject of discussion. One possibility is that the cumulative effects of microevolutionary processes within species give rise to the phenotypic diversity seen among species. Here we showed that when studying the root growth dynamics in root architecture, natural variants of *M. haageana* partially recapitulate the breath of diversity observed in a set of *Mammillaria* species at two different evolutionary time-scales. This might be because, as species evolve and diversify, their natural variants explore the phenotypic space, often reaching the same phenotypic spaces from other species, but also providing the grounds for diversification and speciation. In other words, the outcome of phenotypic microevolution partially recapitulates the patterns generated at the macroevolutionary level, in root architecture in *Mammillaria* species.

Understanding morphological variation is often focused on phenotypes that are relatively easy to observe. Despite being one half of the plant and having relevance for plant nutrition and establishment, plant roots are often overlooked. In cacti and other succulent species this is particularly important because plant roots are an essential organ in charge of foraging water and nutrients which are often scarce in their environments. Here we presented one of the first surveys of root development at different evolutionary time-scales. What remains to be studied is how and why these root phenotypic growth patterns have originated, either driven by stochastic or adaptive evolution, and what genomic footprints these processes have left behind. In this sense, we have started these studies at the genetic and phenotypic level (Hinojosa-Alvarez et al., 2020; Cervantes et al., 2021), which complement other studies on the population genetics and genomic constitution of *Mammillaria* species (Solórzano et al., 2014, 2019; Solórzano and Dávila, 2015; Chincoya et al., 2020). Our results indicate that there is ample variation within a single species, *M. haageana*, which is also present in a range of environments, allowing us to further study the associations between root phenotypes and their environmental conditions of origin. However, this also raises issues about the possible links that can be detected when studying associations of species with the environment, because it is usual (as was our case in non-*M. haageana* species) to take a single accession to represent the entire species, leaving aside the natural variation within each species.

According to our data, the root of *Mammillaria* species is short, because the length of the principal root does not exceed 80 mm in our evaluated time-frame. However, lateral and basal root branches are generated from the principal root, and some species also develop adventitious roots, leading us to think that the root system is shallow and extends horizontally. In fact, it has been proposed that the basal and adventitious roots in *E. platyacanthus* might play an important role during early growth of seedlings under salt stress (Rosas et al., 2021). It has been reported that in other cacti species the root system extends over the most superficial layers of the soil, and this might be an adaptation that allows roots to absorb rainwater quickly (Nobel, 1977; Gulmon et al., 1979; Hunt and Nobel, 1987; Niklas et al., 2002). In other plant species, it has been proposed through mathematical models and experimental validation, that genotypes with shallow and horizontally extended root systems improve the absorption of nutrients such as phosphorus, which has restricted mobility across the soil layers (Heppell et al., 2015; Camilo et al., 2021). Thus, we think that the *Mammillaria* (and perhaps other cacti) root system growth, might display strategies to cope with water stress and low phosphorus soils.

Our phenotypic characterization of root architecture growth was performed in controlled environmental conditions in order to minimize the environmental effects on the phenotype. This meant that our results might have interpretation limitations regarding our experimental environment, and perhaps these types of approaches should be done in multiple environments. However, jumping to a natural or semi natural condition poses a different set of limitations as previously shown in model species (Wilczek et al., 2009; Richards et al., 2012). Further research should be done to attempt bridging the gap between the lab and natural environments. Finally, in the wake of climate change and imminent prolonged droughts, we urge the community to draw more attention toward understanding drought tolerant plants such as cacti, and particularly roots in succulent plants, as this key organ might hold novel insights into water harvesting.

## Data Availability Statement

The datasets presented in this study can be found in online repositories. The names of the repository/repositories and accession number(s) can be found in the article/Supplementary Material.

## Author Contributions

JG-S, IS-S, JL-G, CC, JR-S, SA, and UR designed the research. SA and UR provided the research funds. JG-S, IS-S, and JL-G performed the experiments. IS-S, CC, SA, and UR collected seeds of *M. haageana* accessions. JR-S and SA provided seeds of non-*M. haageana* species. PV determined the identity of fungal contamination. JC-L and JG-S performed statistical analyses. JG-S, IS-S, JL-G, JC-L, and UR prepared the figures. UR and JG-S wrote the manuscript. JG-S, JC-L, and CC edited the manuscript. All authors contributed to the article and approved the submitted version.

## Funding

This work was supported by UNAM-PAPIIT IN211319 to UR. JG-S and JC-L are MSc students, and CC is a doctoral student, all from Posgrado en Ciencias Biológicas, Universidad Nacional Autónoma de México (UNAM), and received the fellowship 1085433, 1084692, and 631251, respectively, from Consejo Nacional de Ciencia y Tecnología (CONACyT-México). This work was done at Laboratorio Nacional de Biodiversidad (CONACyT-México), grant 268109 to UR.

## Conflict of Interest

The authors declare that the research was conducted in the absence of any commercial or financial relationships that could be construed as a potential conflict of interest.

## Publisher's Note

All claims expressed in this article are solely those of the authors and do not necessarily represent those of their affiliated organizations, or those of the publisher, the editors and the reviewers. Any product that may be evaluated in this article, or claim that may be made by its manufacturer, is not guaranteed or endorsed by the publisher.
