# Supplementary material for: Growth Patterns in Seedling Roots of the Pincushion Cactus Mammillaria Reveal Trends of Intra- and Inter-Specific Variation
Source: Front Plant Sci. 2021 Oct 8;12:750623. doi: 10.3389/fpls.2021.750623 (PMC8531529; doi:10.3389/fpls.2021.750623)
Supplement: Supplementary file 2 [file Data_Sheet_2.docx]

Supplementary Material

# Supplementary Figure

***
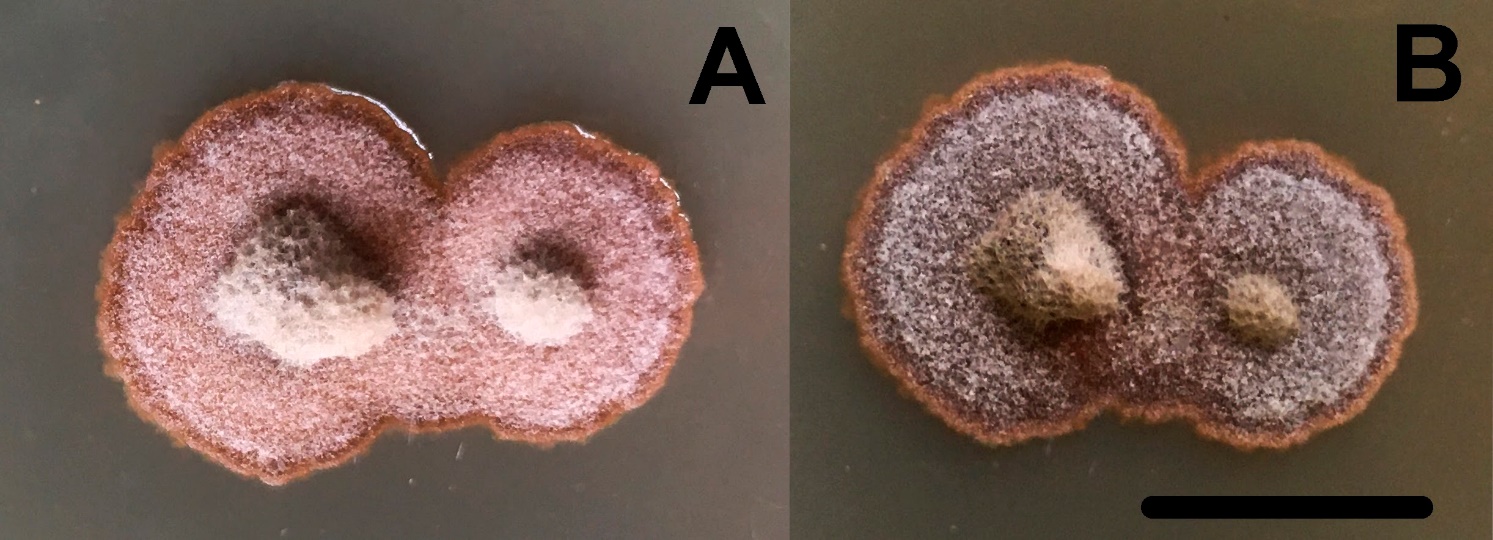
***

**Supplementary Figure 1.** Fungal isolates obtained from germinating seeds of *Mammillaria haageana* growing on potato dextrose agar medium at room temperature after 7 days (A) and 14 days (B). Bar = 10 mm.


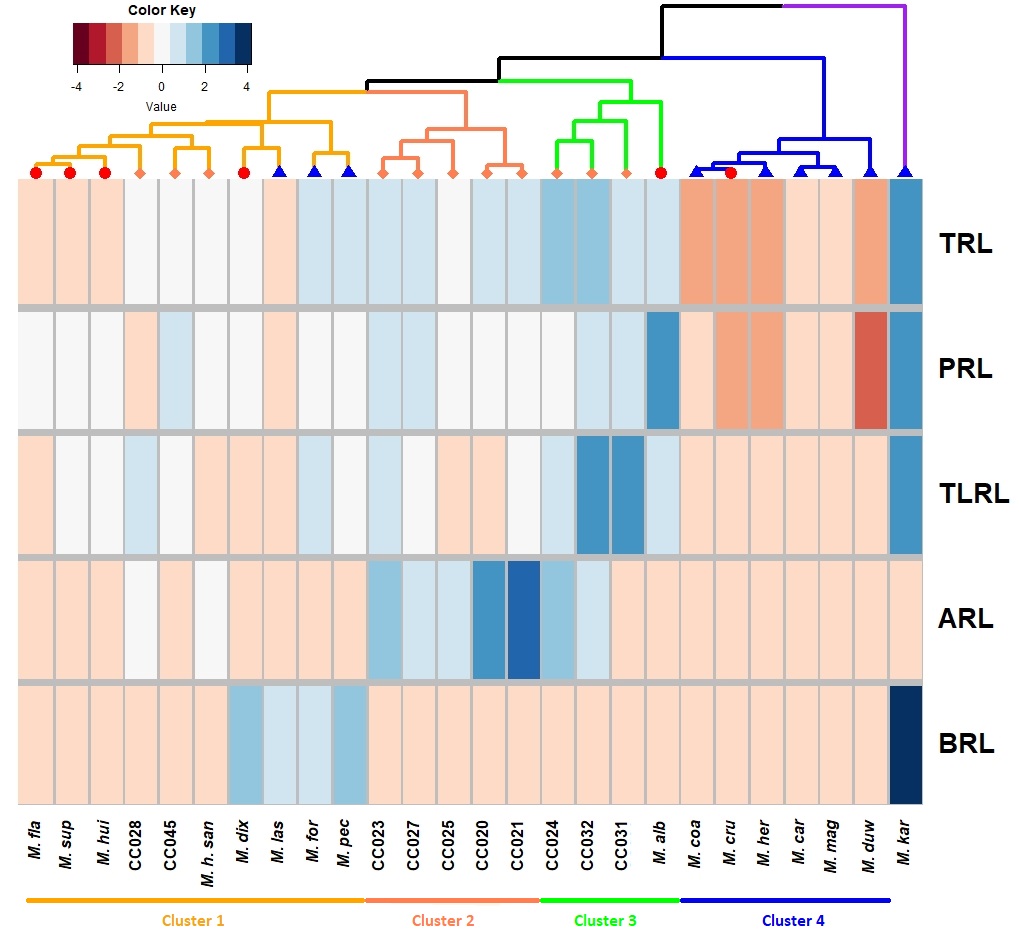


**Supplementary Figure 2.** Clustering (Euclidean distance) analysis using five root variables of *Mammillaria* accessions according to their root architecture phenotypes at 129 days after germination developmental stage. On dendrogram, coral rhomboids represent *M. haageana* accessions, red circles represent *Supertextae* series species, and blue triangles represent non-*Supertextae* series species. TRL: Total Root Length; PRL: Principal Root Length; TLRL: Total Lateral Root Length; ARL: Adventitious Root Length; BRL: Basal Root Length.


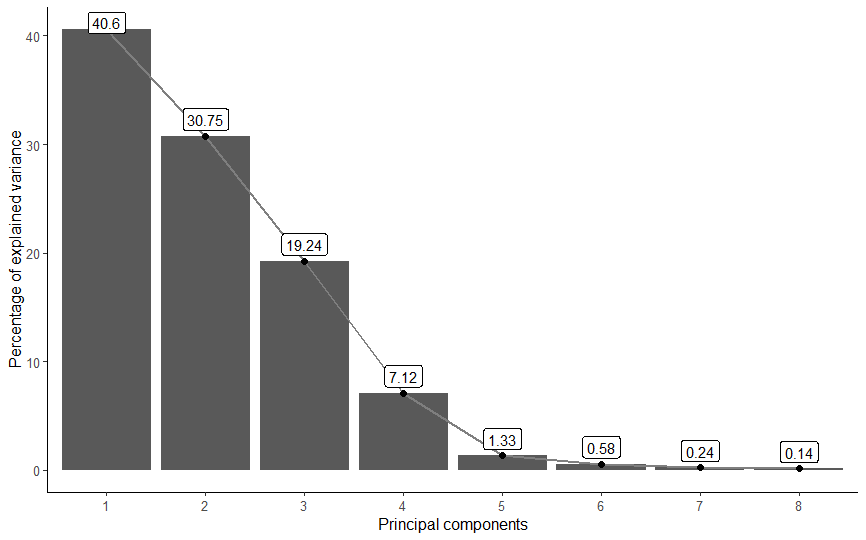


**Supplementary Figure 3.** Variance explained by each of the principal components (PCs). The first three components explain the 90.6% of the variation.

# Supplementary Table

| **Taxonomic level** | **Genotype** | **Number of plants** | **Number of plates** |
| --- | --- | --- | --- |
| ***M. haageana***  **accessions** | CC020, CC021,CC023,CC024,CC028,CC032,CC045 | 30 | 3 |
|  | CC025,CC027 | 27 | 3 |
|  | CC031 | 29 | 3 |
|  | *M.h.san* | 22 | 3 |
| ***Supertextae***  ***Mammillaria* species** | *M. alb, M.cru, M.dix, M.hui, Msup* | 30 | 3 |
|  | *M. fla* | 40 | 3 |
| **non-*Supertextae***  ***Mammillaria***  **species** | *M.car* (*Polyedrae*)*. M.coa* (*Mammillaria*)*. M.for* (*Leucocephalae*)*. M.her* (*Longiflorae*)*. M.kar* (*Polyedrae*)*. M.mag* (*Mammillaria*)*. M.pec* (*Pectiniferae*)*.* | 30 | 3 |
|  | *M. duw* (*Stylothelae*)*. M.las* (*Lasiacanthae*)*.* | 20 | 2 |

**Supplementary Table 1.** Number of individual plants used in each of the *Mammillaria* accessions, *Supertextae* series species, and non-*Supertextae* series species. Number of plates used in each species. Each plate contained 10 individuals. The third plate of accessions CC025 and CC027 contained seven individuals, as well as CC031 and *M. h. san* contained nine and two individuals respectively.

| **Root**  **Attributes** | **PC1** | **PC2** | **PC3** | **PC4** | **PC5** | **PC6** | **PC7** | **PC8** |
| --- | --- | --- | --- | --- | --- | --- | --- | --- |
| TRL | 0.8499 | 0.0564 | 0.1375 | 0.0001 | 0.1119 | 0.0049 | 0.0257 | 0.0450 |
| PRL | 0.4625 | 0.0706 | 0.0010 | 0.4075 | 0.0674 | 0.0052 | 0.0048 | 0.0179 |
| nLR | 0.6236 | 0.0182 | 0.1196 | 0.1944 | 0.0008 | 0.0728 | 0.0021 | 0.0004 |
| TLRL | 0.6678 | 0.0526 | 0.1180 | 0.1083 | 0.0768 | 0.0584 | 0.0060 | 0.0000 |
| nAR | 0.1662 | 0.3823 | 0.3224 | 0.0211 | 0.0012 | 0.0025 | 0.1103 | 0.0003 |
| ARL | 0.2524 | 0.3378 | 0.3217 | 0.0093 | 0.0864 | 0.0108 | 0.0722 | 0.0073 |
| nBR | 0.0001 | 0.6166 | 0.2804 | 0.0009 | 0.0024 | 0.0003 | 0.0021 | 0.1184 |
| BRL | 0.0097 | 0.6149 | 0.3250 | 0.0074 | 0.0772 | 0.0025 | 0.0074 | 0.0862 |

**Supplementary Table 2.** Communality of proportion of variance explained by each variable within each principal component (PC).

| **Level** | **Specie** | **PC1** | **PC2** | **PC3** |
| --- | --- | --- | --- | --- |
| *M. haageana* | CC020 | abc | a | a |
| *M. haageana* | CC021 | abc | ab | a |
| *M. haageana* | CC023 | abcd | bc | bcdef |
| *M. haageana* | CC024 | a | bc | bcdgh |
| *M. haageana* | CC025 | defg | bc | bcdgh |
| *M. haageana* | CC027 | abcd | bc | bcdeg |
| *M. haageana* | CC028 | def | cde | efijk |
| *M. haageana* | CC031 | abcd | df | ijk |
| *M. haageana* | CC032 | ab | ce | bcefij |
| *M. haageana* | CC045 | cdef | gh | bcdeg |
| *M. haageana* | *M.h.san* | cdefg | ab | aghl |
| *Supertextae* | *M.alb* | bcde | gi | ijk |
| *Supertextae* | *M.cru* | hij | ce | bcefi |
| *Supertextae* | *M.dix* | hij | df | bcefi |
| *Supertextae* | *M.fla* | h | bc | efijk |
| *Supertextae* | *M.hui* | gkl | df | jk |
| *Supertextae* | *M.sup* | jkl | dfi | fijk |
| Non-*Supertextae* | *M.car* | hi | def | bcef |
| Non-*Supertextae* | *M.coa* | fg | df | k |
| Non-*Supertextae* | *M.duw* | efgk | gh | al |
| Non-*Supertextae* | *M.for* | jkl | df | befijk |
| Non-*Supertextae* | *M.her* | h | ce | bcdeg |
| Non-*Supertextae* | *M.kar* | a | h | ahl |
| Non-*Supertextae* | *M.las* | ijkl | fgi | cdgh |
| Non-*Supertextae* | *M.mag* | ijl | dfi | bcdegh |
| Non-*Supertextae* | *M.pec* | fgk | g | dghl |

**Supplementary Table 3.** Kruskal-Wallis test showed significant differences in the first three components (PC1=H(2):431.12, *p<*0.0001; PC2=H(2):335.11, *p<*0.0001; PC3=H(2):189.45, *p<*0.0001). Dunn test (Post-hoc test for non-parametric data) on the first three principal components, where the groups that do not share letters are significantly different (*p*<0.05).
